# Diet Composition, Anthropometrics, and Mortality Risk

**DOI:** 10.3390/ijerph191912885

**Published:** 2022-10-08

**Authors:** Nir Y. Krakauer, Jesse C. Krakauer

**Affiliations:** 1 Department of Civil Engineering, City College of New York, New York, NY 10031, USA; 2 Associated Physicians/Endocrinology, Berkley, MI 48072, USA

**Keywords:** ARIC, obesity, diet, risk assessment, anthropometry, body shape

## Abstract

While overeating is considered a cause of the obesity epidemic as quantified by body mass index (BMI), the association of diet with a body shape index (ABSI) and hip index (HI), which are transformations of waist and hip circumference that are independent of BMI and which predict mortality risk, is poorly known. We used data from the Atherosclerosis Risk in Communities (ARIC) study of about 15,000 middle-aged adults to investigate associations between macronutrient intake (energy, carbohydrate, protein, and fat, the latter two divided into plant and animal sources, all based on self-reported food frequency) with anthropometric indices (BMI, ABSI, and HI). We also analyzed the association of diet and anthropometrics with death rate during approximately 30 years of follow-up. High intake of energy and animal fat and protein was generally associated with higher ABSI and lower HI at baseline, as well as greater mortality hazard. BMI was also positively linked with animal fat and protein intake. In contrast, higher intake of carbohydrates and plant fat and protein was associated with lower ABSI and BMI, higher HI, and lower mortality hazard. For example, after adjustment for potential confounders, each standard deviation of additional plant fat intake (as a fraction of total energy) was associated with a 5% decrease in mortality rate, while animal fat intake was associated with a 5% mortality increase per standard deviation. The directions of the associations between diet and anthropometrics are consistent with those found between anthropometrics and mortality without reference to diet.

## 1. Introduction

Connections between diet and health have been recognized since ancient times [1]. Increased consumption of energy-dense, often highly processed foods contributes to increased incidence of overweight and obesity and frequency of ‘lifestyle’ diseases, including diabetes, heart disease, and cancer [2]. Calorie restriction is a promising intervention that substantially delays aging in a variety of smaller animals, including rodents and primates, although the effects in humans are still uncertain [3].

Moreover, the benefits of calorie restriction may be a function not so much of total food energy intake, but of the intake of specific macronutrients. For example, low-protein high-carbohydrate diets extend lifespan in laboratory-grown insects and rodents, with the optimal macronutrient ratio being similar to that consumed by some long-lived human populations [4,5]. In fish, intermediate lipid intake in males and low protein intake in females resulted in the lowest mortality rates [6].

Plant and animal sources of macronutrient may also have distinct human health impacts. For example, in the United States Third National Health and Nutrition Examination Survey, respondents aged 50 to 65 reporting high animal protein intake experienced higher all-cause and cancer mortality rates on follow-up, an association not seen for plant protein intake [7]. Animal protein intake, particularly from meat, fish and shellfish, was associated with increased risk of both global and abdominal obesity in the Observation of Cardiovascular Risk Factors in Luxembourg study [8]. In the Nurses’ Health Study, Health Professionals Follow-up Study, and National Institutes of Health -AARP Diet and Health Study, reporting plant rather than animal protein intake was associated with lower all-cause and cardiovascular mortality [9,10].

The connection between diet and weight, or more commonly weight allometrically adjusted for height to yield body mass index (BMI), has been intensively studied. Total energy intake has been connected to increasing rates of overweight and obesity [11,12]. The roles of carbohydrates versus fat in promoting obesity and associated disease have also featured in a continuing controversy [13]. However, associations between diet and other body dimensions are less clear.

Waist and hip circumferences (WC and HC) can be allometrically normalized for height and weight, respectively yielding a body shape index (ABSI) and hip index (HI) [14,15]. As the ‘new anthropometrics’ [16], ABSI and HI have been shown to predict mortality risk independently of BMI, and are also associated with cancer development and a variety of metabolic and endocrine biomarkers [17,18,19,20,21,22]. Because BMI, ABSI, and HI are statistically uncorrelated (with correlations between them in populations typically being of order 0.1 or less), the risks associated with each combine to produce a more accurate individualized risk assessment, which can be expressed as an anthropometric risk index (ARI) [15]. Yet, there are only a few studies relating diet to ABSI or HI. Two such studies showed that lower food security score in the Indonesia Family Life Survey was associated with higher ABSI and systolic blood pressure [23] and that teams of obese New Zealand Pacific and Māori people who met weight loss challenges showed reduction in ABSI [24].

Here, we investigate the associations between diet and the new anthropometrics ABSI and HI, as well as mortality risk, in a large general population sample, the Atherosclerosis Risk in Communities (ARIC) study [25]. The aims of the current work are as follows: (1) to assess associations with ABSI and HI (and their complement BMI) of diet parameters related to macronutrient consumption; and (2) to assess whether mortality risk associated with anthropometric and diet parameters could be mediated by the association of diet with anthropometrics.

## 2. Methods

### 2.1. Data

ARIC is a long-term prospective study of approximately 15,000 initially middle-aged American adults, who were surveyed and examined initially in the late 1980s (1986–1990) and then at 3-year intervals. We used release v2021a of the study data, which includes follow-up for for mortality, heart disease, and stroke events through 2017 (27–31 years since enrollment), as obtained from the National Heart, Lung, and Blood Institute Biologic Specimen and Data Repository Information Coordinating Center. Diet data were self-reported at the initial examination in a 66-item semi-quantitative food frequency questionnaire, which was converted by ARIC investigators into estimated nutrient intakes using the Harvard Nutrient Database [26]. Anthropometrics (height, weight, WC, and HC) were measured at the initial examination by trained examiners. The ARIC data include information on deaths identified based on follow-up telephone calls and linkage to local hospital and state health department records and the National Death Index. We analyzed the association between diet and anthropometric data collected at enrollment (1986–1990), on the one hand, and mortality on follow-up through 2017, on the other hand. Other demographic, behavioral, and medical history information from the initial visit was also considered as possibly confounding the relationship between diet, anthropometric, and mortality.

The ARIC protocol was approved by the University of North Carolina at Chapel Hill Office of Human Research Ethics, and all participants gave written informed consent [25]. The present analysis of already-anonymized public-use data from ARIC was approved by the University Integrated Institutional Review Board of the City University of New York (file number 2015-0768, letter dated 6 July 2021).

### 2.2. Anthropometric and Diet Measures

As in previous work [15], anthropometrics were converted into indices that are approximately mutually uncorrelated, as follows:

(1) $$\mathrm{BMI}\equiv W\cdot H^{-2},$$

(2) $$\mathrm{ABSI}\equiv \mathrm{WC}\cdot H^{5/6}\cdot W^{-2/3},$$

(3) $$\mathrm{HI}\equiv \mathrm{HC}{\left(\frac{H}{\langle H\rangle }\right)}^{0.310}{\left(\frac{W}{\langle W\rangle }\right)}^{-0.482},$$

H refers to height and W to weight, and 〈H〉= 166 cm and 〈W〉= 73 kg were typical values.

In turn, these indices were nondimensionalized using z-score transformations [14,27]:

(4) $$\mathrm{zI}\equiv \frac{I-\langle I\rangle }{\sigma_{I}},$$

where I denotes an index, zI is its transformed value, 〈I〉 is its average value in ARIC for the individual’s age and sex, and $\sigma_{I}$ is its sex- and age-specific standard deviation in ARIC. This transforms each index to have mean near 0 and standard deviation near 1.

The BMI, ABSI, and HI log hazard ratios estimated by Cox proportional hazard models with a smoothing spline basis were summed to give anthropometric risk index (ARI) values that estimate the overall relative log mortality hazard associated with anthropometrics for each individual [15]. ARI is the sum of three nonlinear functions—one each for BMI, ABSI, and HI z score—which sums the contribution each makes to the mortality hazard. ARI near zero suggests the average mortality hazard given the individual’s age and sex (since $e^{0}=1$), while, for example, ARI near 0.7 suggests that the individual’s expected death rate is approximately twice the average (since $e^{0.7}\approx 2$).

The diet attributes considered were total energy intake (in kcal); macronutrient intakes of carbohydrate, protein and fat, and subdivisions of protein and fat into animal and plant (or vegetable) sources. The carbohydrate, fat, and protein intakes, as well as their plant and animal shares, were all expressed as fractions of total energy, and so ranged between 0 and 1. All the diet measures were nondimensionalized in the same way as the anthropometric indices by z-score transformation using the ARIC age and sex specific averages and standard deviations.

### 2.3. Statistical Analysis of Associations

We constructed linear models for the association of each diet measure z score (as predictor) with each anthropometric index z score (as predicand). We also constructed and plotted smoothing spline fits (with smoothing parameter determined by leave-one-out cross validation) to check for nonlinearity in these associations.

Associations of each anthropometric index z score with mortality were determined using Cox proportional hazard modeling with age as the timescale (the results of the analysis were used to determine ARI values for each individual in the study cohort). Associations of each diet measure z score with mortality were similarly determined using Cox proportional hazard modeling. The coefficient from the Cox proportional hazard model that quantifies the association between each diet measure and log mortality hazard was compared to that from linear regression quantifying the association between each diet measure and ARI to assess approximately what fraction of the association of the diet measure with mortality is mediated via the association of diet with simple anthropometrics and, in turn, their association with mortality, as captured by ARI.

In order to check for robustness to confounders, these analyses were repeated for models, where additional variables from the ARIC initial survey that were found to affect mortality risk were included as predictors of the anthropometric or mortality outcome. These adjusted models also included as linear predictors sex, race, income, years of education, smoking (categorized as never, former, or current smoker), alcohol consumption, previous diabetes diagnosis, previous asthma diagnosis, physical activity indices (including work, sports, and other leisure activity), and hormone replacement therapy in women.

We computed as a measure of model quality for the different mortality prediction models the Akaike information criterion reduction (ΔAIC). The reduction in the Akaike information criterion score is given relative to a null model with no predictors (for the unadjusted analyses) or to a model without the diet predictor (for the adjusted analyses). Higher ΔAIC indicates that a model fits the data better, accounting for the model complexity. A difference in ΔAIC of about 6 indicates that the model with higher value is likely at the 95% confidence level to be the better predictor of mortality [28,29].

Analyses were conducted in the R programming language, version 4.1.2 [30]. Cox proportional hazard modeling for mortality risk was carried out using the coxph function of the survival package, version 3.2.13 [31,32].

## 3. Results

### 3.1. Cohort Characteristics

Data from 13714 individuals who had valid values for anthropometrics, diet composition, the other variables adjusted for, and mortality follow-up, were included in this study. Of these, 7444 (54%) were female. The median initial age was 54 (range: 44–66). In total, 76% were White and the remainder Black. During follow-up, 6934 deaths occurred.

Median energy intake (with interquartile range) was 1530 (1190–1960) calories per day. The median energy fractions from each macronutrient were 48.5% (42.6–54.7) carbohydrate, 17.8% (15.1–20.4) protein (4.3% (3.6–5.1) vegetable protein and 13.2% (10.6–16.0) animal protein), and 33.2% (28.6–37.4) fat (12.5% (9.3–16.0) vegetable fat and 19.8% (15.7–23.9) animal fat). Values for anthropometrics were 26.8 (24.0–30.3) kg m${}^{-2}$ for BMI, 0.0823 (0.0792–0.0852) m${}^{11/6}$ kg${}^{-2/3}$ for ABSI, and 101.7 (97.6–106.3) cm for HI.

### 3.2. Anthropometrics Associations with Mortality

**Figure 1:**
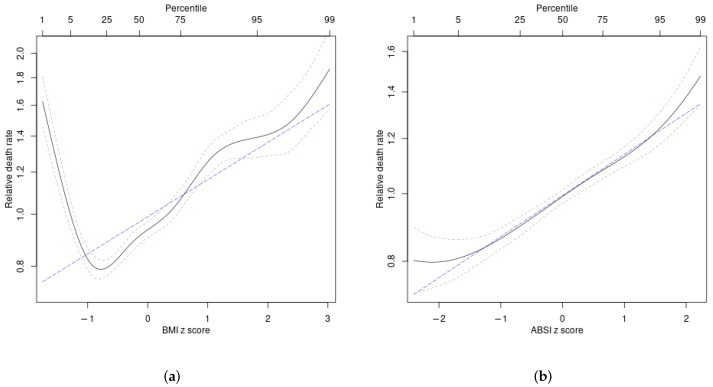

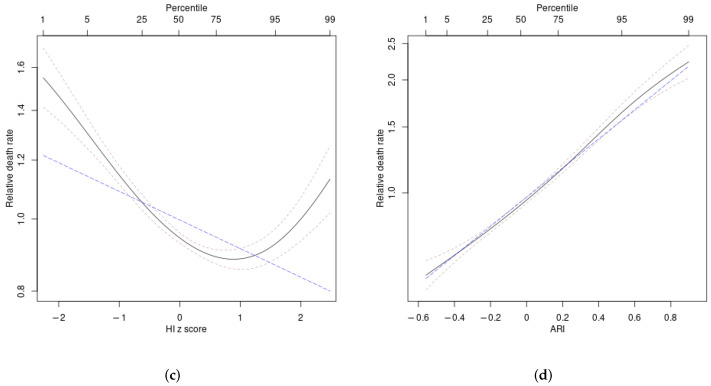
Estimated mortality hazard ratios in ARIC as nonlinear (penalized spline) functions of (**a**) BMI, (**b**) ABSI, (**c**) HI, (**d**) ARI (solid curves). Thin dashed curves indicate 95% confidence intervals. The blue long-dashed line shows the corresponding best linear fit, which can be seen in some cases to not capture well the underlying nonlinear relationship.

Associations of anthropometrics with mortality, used to compute ARI, are shown in Figure 1. Mortality hazard is lowest for BMI somewhat below average, increasing for both very low and high BMI. Mortality hazard is lowest for HI somewhat below average, increasing for both low and very high HI. Mortality hazard increases monotonically with increasing ABSI. The logarithm of mortality hazard is nearly linearly proportional to ARI, showing that it is a function of anthropometrics that captures well their joint contribution to mortality hazard.

### 3.3. Diet Associations with Anthropometrics

Table 1 shows the linear regression coefficients between diet and anthropometrics z scores, as well as ARI. We consider each diet attribute in turn, starting with the unadjusted regression results and noting where the result is different with adjustment for potential confounders.

Total energy intake is not significantly associated with BMI in the unadjusted analysis (but does show a significant positive association with BMI in the adjusted analysis). Energy intake shows a significant direct relationship with ABSI and inverse relationship with HI. Overall, the anthropometric profile associated with high reported energy intake is a more hazardous one (higher ARI).

Fraction of carbohydrate correlates negatively with BMI and ABSI and positively with HI. After adjustment for covariates, there is no significant association with ABSI. For the unadjusted regression, smoothing splines show a steeper decrease of ABSI with carbohydrate fraction for below-average values, leveling off at above-average carbohydrate fractions; by contrast, the associations with BMI and HI look linear (not shown). Overall, higher carbohydrate fraction correlates with lower anthropometric mortality risk as expressed by ARI.

Fraction of protein correlates positively with BMI, negatively with ABSI, and not at all with HI. The negative association with ABSI is not significant after adjustment for confounders. Associations differ between plant protein and animal protein. While AP is correlated positively with BMI, the association of VP with BMI is negative. Animal protein shows a more negative association with ABSI than plant protein in the unadjusted analysis, but not after adjustment. Plant protein shows a positive association with HI, while the association of HI with animal protein is negative. ARI shows no association with total protein fraction in the unadjusted analysis, but a positive association in the adjusted analysis. Both analyses show that more plant protein intake is associated with lower ARI while more animal protein intake is associated with higher ARI.

Fraction of fat correlates positively with BMI. It has a significant positive correlation with ABSI only in the unadjusted analysis, and a significant negative association with HI only in the adjusted one. Animal and vegetable fat sources show contrasting associations. Plant fat is negatively associated with BMI, negatively associated with ABSI (adjusted analysis only), and positively associated with HI (unadjusted analysis only). Animal fat shows opposite associations, being positively correlated with both BMI and ABSI and negatively correlated with HI. Anthropometric risk, as expressed by ARI, increases for higher fractions of energy from total fat and animal fat, but decreases for higher fraction from plant fat.

### 3.4. Diet Associations with Mortality

The association of each diet z score as a linear predictor of log mortality hazard, estimated using Cox proportional hazard modeling, is presented in Table 2. More total energy and animal fat is associated with higher mortality hazard, while more carbohydrate and plant fat is associated with lower mortality hazard. For protein, adjustment for confounders has a substantial impact: in the unadjusted analysis, more plant protein is associated with lower mortality, while in the adjusted analysis, this association is no longer significant, but instead total and animal protein are both associated with higher mortality. To give a sense of the magnitude of the associations given in Table 2, each standard deviation increase in plant fat is associated (after adjustment for confounders) with an approximately 5% lower mortality rate ($1-e^{-0.052}$), while each standard deviation increase in animal fat is associated (after adjustment for confounders) with an approximately 5% higher mortality rate ($e^{0.047}-1$).

The association of diet with mortality is largely consistent in the sign with the association of diet with ARI: higher energy intake and animal fat calorie fraction are associated with greater mortality hazard, while higher carbohydrate, plant protein, and plant fat calorie fractions are associated with lower mortality hazards. However, the magnitudes of the regression coefficients with log mortality hazards tend to be roughly 3 times larger than those of ARI, indicating that only a minority of the association of diet with mortality is mediated through the association of diet with basic anthropometric measurements and, in turn, the association of these anthropometrics with mortality.

**Figure 2:**
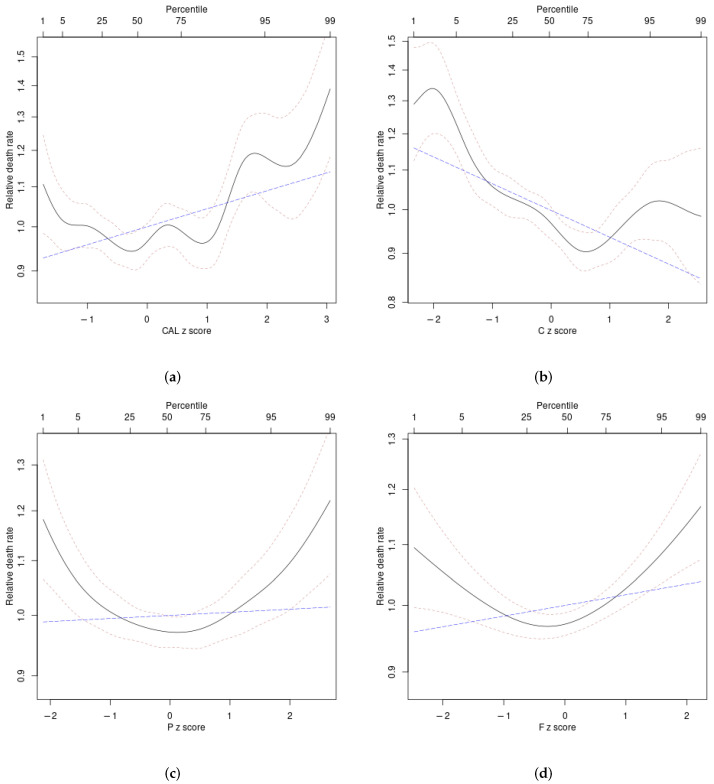
Estimated mortality hazard ratios in ARIC as nonlinear (penalized spline) functions of (**a**) energy intake (CAL), (**b**) carbohydrates (C), (**c**) protein (P), (**d**) fat (F) (solid curves). Thin dashed curves indicate 95% confidence intervals. The blue long-dashed line shows the corresponding best linear fit, which can be seen in some cases to not capture well the underlying nonlinear relationship.

**Figure 3:**
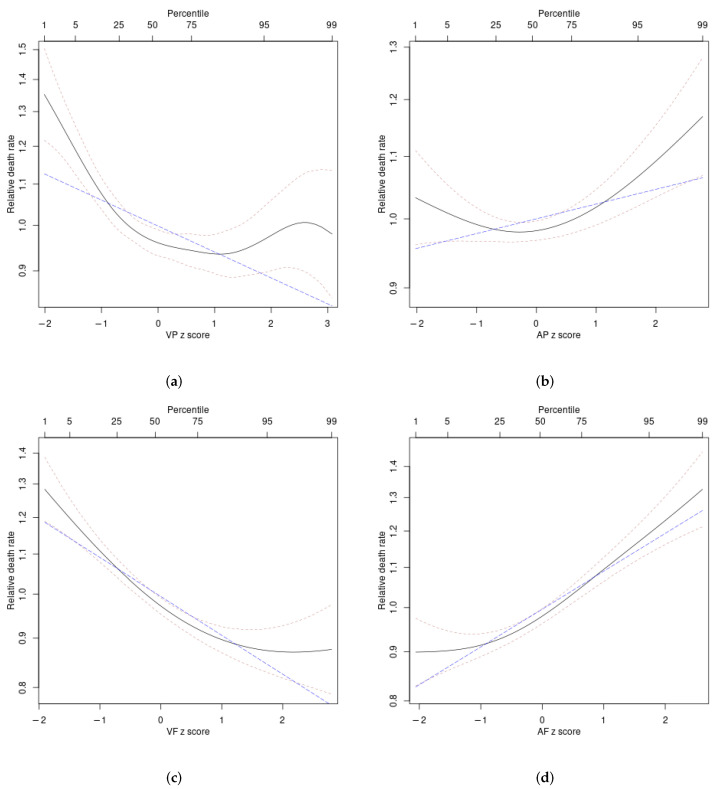
Estimated mortality hazard ratios in ARIC as nonlinear (penalized spline) functions of fraction of calories from vegetable and animal sources: (**a**) plant protein (VP), (**b**) animal protein (AP), (**c**) vegetable fat (VF), (**d**) animal fat (AF) (solid curves). Thin dashed curves indicate 95% confidence intervals. The blue long-dashed line shows the corresponding best linear fit, which can be seen to in some cases not capture well the underlying nonlinear relationship.

Nonlinear (smoothing spline) associations of the diet z scores with mortality hazard (unadjusted analyses) are shown in Figure 2 and Figure 3. For energy intake, mortality hazard was elevated only at considerably above-average values (z score above 1). Mortality hazard was also elevated at below-average values (negative z score) of carbohydrate fraction. Protein and fat fractions both showed U-shaped associations with mortality hazard, with near-average values associated with the lowest risk (Figure 2). For protein, high hazard is primarily associated with below-average vegetable protein and above-average animal protein intake. For fat, hazard increases fairly monotonically with lower vegetable fat and higher animal fat intake (Figure 3).

The difference in ΔAIC between each linear and nonlinear analysis in Table 2 provides a quantitative measure of the nonlinearity in the relationships between each diet z score and log mortality hazard. For the unadjusted models, all the associations, except that for animal fat, were significantly nonlinear (with ΔAIC for the nonlinear models more than 6 greater than that for the linear models), consistent with Figure 2 and Figure 3. For the adjusted models, nonlinearity seems to be less important.

## 4. Discussion

Overall, for the first time in a large general-population sample, we find significant associations of diet components (including total energy and macronutrients) with ABSI and HI, as well with BMI. Further, there are differences in the association with anthropometrics by macronutrient source (plant versus animal) for protein and fat. The associated anthropometric profile for higher intake of total energy, animal protein, and animal fat is somewhat more risky (as quantified using ARI), whereas that for higher intake of carbohydrates, plant protein, and plant fat is somewhat less so. Modeling the association between diet attributes and mortality directly gives very similar results. Further, these associations held in models that adjusted for other baseline risk factors that could be potential confounders, including demographics, medical conditions, and behaviors, such as smoking, alcohol consumption, and physical inactivity.

Our results are consistent with previous analyses of the separate associations of anthropometrics and diet with mortality in ARIC (with fewer years of follow-up). Ref. [15] found that the association of mortality hazard with BMI and HI showed U shapes, whereas mortality hazard increased monotonically with increasing ABSI. Ref. [26] found that even after adjustment for covariates, low carbohydrate intake was associated with higher mortality in ARIC, primarily when accompanied by high intake of animal protein and fat. Our main contribution here is to show that these diet factors are also associated with the anthropometrics ABSI and HI (as well as BMI), and in a direction that partly explains the association of diet factors with mortality. Higher carbohydrate intake and lower animal protein and fat intake was a dietary pattern associated with lower BMI and ABSI and higher HI, as well as lower mortality hazard.

A number of limitations should be kept in mind when evaluating the findings presented here. While we only examined the association with anthropometrics and mortality hazard of total calorie intake and macronutrient composition (along with plant/animal split), other aspects of diet quality, including vitamin, phytochemical, and mineral intakes, are also likely to have substantial associations with anthropometrics and health outcomes [33]. Within protein intake, certain amino acids may be particularly important in modulating aging-related metabolic and molecular pathways [34,35]. Studies have distinguished between healthy plant food and unhealthy plant food (such as sugary drinks and juices, fried and salted potatoes, and refined grains), finding that the latter is associated with higher risk of metabolic syndrome and coronary heart disease [36]. In general, the consumption of ultra-processed foods is associated with higher mortality risk [37]. Many of these aspects could be further examined using the food frequency data available in ARIC. Additionally, associations of nutrition may be dependent on age—for example, in mice, the optimal lifespan was obtained with a diet high in carbohydrates in early and middle life, but higher in protein during old age [7,38]. ARIC is not ideally suited to study such variability, as all participants were middle-aged at recruitment.

A limitation of ARIC, along with most other large cohort studies, is that that dietary intakes were self-reported. The median reported daily intake was 1530 calories (mean: 1630). This is low compared to national-level computations based on the quantities of food supplied adjusted for estimated losses, which yield mean intakes of over 2500 calories per day [39,40]. The low correlation of BMI with energy intake is also unexpected, given that direct measurement methods (e.g., calorimetry and doubly labeled water) clearly show that energy expenditure tends to be greater in adults with higher BMI [41]. Indeed, studies find that adults with higher BMI disproportionately under-report their food intake in both food frequency questionnaires and 24 h food intake recalls [42]. In general, it is expected that measurement error and bias of self-reported dietary intakes would tend to attenuate associations between diet and objectively measured aspects of health (including both anthropometrics and death) [43]. Self-reported nutrient intakes as fractions of total calories, as considered here, are found to be more reliable than absolute values, and with cautious interpretation, relating self-reported dietary data to important health outcomes continues to be of value in epidemiology [44].

Even aside from measurement error and bias, population associations observed in cohorts such as this ARIC cannot prove the causal effect of diet on anthropometrics or mortality, which ideally could be established through long-term randomized controlled trials. For example, a meta-analysis of randomized controlled trials showed that eating a smaller proportion of energy from fat has a small weight-reducing effect in adults [45], which supports a causal component for the positive association found in ARIC of energy fraction from fat with BMI. Further, the White and Black USA ARIC cohort reported quite a high consumption of animal fat and protein by world standards, and the dietary associations seen in it may not apply to other nationalities and subpopulations with different diet and lifestyle patterns. Moreover, different diet components are inter-correlated and also associated with other potentially confounding traits and behaviors [46]. Principal component analysis and cluster analysis of the intake of different nutrients and food categories can be used to identify diet patterns within the population that correlate with biological pathways related to health and can be used to formulate readily understandable dietary advice [47,48]. Diet quality indices offer one avenue to consider sets of diet components that may together contribute to well-being [49,50].

Despite caveats, in conjunction with other types of data, the associations seen in prospective studies such as ARIC help inform hypotheses about the role of diet in health and the potential mediation of anthropometrics. Diet intervention studies could consider measuring change in anthropometrics, including ABSI and HI, and the associated anthropometric risk estimate (ARI) calibrated using appropriate cohorts, as one intervention outcome, which would allow more definitive statements to be made about causality without requiring many years of follow-up for morbidity and mortality.

## 5. Conclusions

So far, there have been few studies of the association of diet attributes with waist and hip circumference-based allometric anthropometrics, ABSI and HI. Here, using self-reported food frequency questionnaire responses in a large middle-aged community cohort, we find that high intake of energy and animal fat and protein is generally associated with higher ABSI and lower HI as well as greater mortality hazard on follow-up. In contrast, higher intake of carbohydrates and plant fat and protein is associated with lower ABSI, higher HI, and lower mortality hazard. The directions of these associations are consistent with those found between anthropometrics and mortality directly, and suggest that some of the association between anthropometrics and mortality hazard could stem from the influence of diet on both.

## Figures and Tables

**Table 1:** Diet associations with anthropometrics.

Diet Attribute		Body Mass Index	A Body Shape Index	Hip Index	Anthropometric Risk Indicator
Energy	Unadjusted	8 ± 8	35 *** ± 9	−25 ** ± 8	15 *** ± 3
	Adjusted	19 * ± 8	18 * ± 8	−23 ** ± 8	12 *** ± 3
Carbohydrate	Unadjusted	−53 *** ± 8	−26 ** ± 9	22 * ± 9	−15 *** ± 3
	Adjusted	−85 *** ± 8	5 ± 9	20 * ± 9	−11 *** ± 3
Protein	Unadjusted	112 *** ± 8	−46 *** ± 9	−2 ± 9	2 ± 3
	Adjusted	99 *** ± 8	−15 ± 9	0 ± 9	10 *** ± 3
Plant protein	Unadjusted	−57 *** ± 8	−10 ± 8	66 *** ± 8	−15 *** ± 3
	Adjusted	−53 *** ± 8	−13 ± 8	55 *** ± 9	−10 *** ± 3
Animal protein	Unadjusted	125 *** ± 8	−43 *** ± 9	−22 * ± 9	7 * ± 3
	Adjusted	109 *** ± 8	−11 ± 9	−17 ± 9	12 *** ± 3
Fat	Unadjusted	70 *** ± 8	27 ** ± 9	−16 ± 9	15 *** ± 3
	Adjusted	62 *** ± 8	1 ± 9	−21 * ± 9	10 *** ± 3
Plant fat	Unadjusted	−50 *** ± 8	7 ± 9	37 *** ± 9	−13 *** ± 3
	Adjusted	−35 *** ± 8	−20 * ± 9	17 ± 9	−11 *** ± 3
Animal fat	Unadjusted	115 *** ± 8	23 ** ± 9	−47 *** ± 9	26 *** ± 3
	Adjusted	91 *** ± 8	18 * ± 8	−35 *** ± 9	19 *** ± 3

Regression coefficients for anthropometrics (±standard error, all multiplied by 1000 to avoid decimals) as a function of diet in the ARIC initial visit. Z score transformations of anthropometrics (except the anthropometric risk indicator) and diet attributes were used in the regression, so each coefficient can be understood as the standard deviation change in each anthropometric index per standard deviation change in a diet attribute. For each diet attribute, the first set of coefficients given (“unadjusted”, colored red) is for linear regression without covariates, and the second (“adjusted”, colored blue) is for a model that also includes demographic, behavioral, and medical history variables. Significance level of coefficients: * 0.05, ** 0.01, *** 0.001.

**Table 2:** Diet associations with mortality.

Diet Attribute		Mortality Hazard	ΔAIC
		Coefficient	(Linear/Nonlinear)
Energy	Unadjusted	42 *** ± 12	10.4/31.3
	Adjusted	37 ** ± 12	7.7/10.9
Carbohydrate	Unadjusted	−68 *** ± 12	31.1/50.3
	Adjusted	−33 * ± 13	4.6/10.5
Protein	Unadjusted	2 ± 12	−2.0/14.3
	Adjusted	41 ** ± 13	8.4/8.6
Plant protein	Unadjusted	−63 *** ± 12	25.6/39.8
	Adjusted	−17 ± 12	−0.1/2.8
Animal protein	Unadjusted	20 ± 12	0.7/11.1
	Adjusted	44 *** ± 13	10.0/16.4
Fat	Unadjusted	19 ± 12	0.3/14.3
	Adjusted	7 ± 13	−1.7/−0.2
Plant fat	Unadjusted	−90 *** ± 12	51.4/61.4
	Adjusted	−52 *** ± 13	15.0/18.3
Animal fat	Unadjusted	90 *** ± 12	52.6/58.1
	Adjusted	47 *** ± 12	12.7/13.0

Relationship of baseline diet attributes with subsequent mortality hazard in ARIC, from Cox proportional hazard modeling. Mortality hazard coefficients represent the increase in logarithm of death rate ± standard error (multiplied by 1000 to avoid decimals) per standard deviation change in each diet attribute, treated as a linear predictor in the Cox model. Reduction in Akaike information criterion (ΔAIC) relative to a model without the diet attributes is given for the diet attribute entered either only as a linear predictor of mortality or also allowing for a nonlinear relationship. For each diet attribute, the first set of coefficients given (“unadjusted”, colored red) is for a model without covariates, and the second (“adjusted”, colored blue) is for a model that also includes demographic, behavioral, and medical history variables. Significance level of hazard coefficients: * 0.05, ** 0.01, *** 0.001.

## Data Availability

The cohort data used in this study are available by application to the National Heart, Lung, and Blood Institute’s Biologic Specimen and Data Repository Information Coordinating Center at https://biolincc.nhlbi.nih.gov/studies/aric/, accessed on 17 August 2022.
